# Mortality, material deprivation and urbanization: exploring the social patterns of a metropolitan area

**DOI:** 10.1186/s12939-015-0182-y

**Published:** 2015-06-09

**Authors:** Paula Santana, Claudia Costa, Marc Marí-Dell’Olmo, Mercè Gotsens, Carme Borrell

**Affiliations:** Departamento de Geografia, Centro de Estudos de Geografia e Ordenamento do Território, Universidade de Coimbra, Colégio S. Jerónimo, Largo D. Dinis, 3000-043 Coimbra Portugal; CIBER Epidemiología y Salud Pública (CIBERESP), 3-5, Pabellón 11. Planta 0, Monforte de Lemos, 28029 Madrid Spain; Agència de Salut Pública de Barcelona, Plaça Lesseps, 1, 08023 Barcelona, Spain; Institut d’Investigació Biomèdica (IIB Sant Pau), Sant Antoni Maria Claret, 167, 08025 Barcelona, Spain; Universitat Pompeu Fabra, Doctor Aiguader, 80, 08003 Barcelona, Spain

**Keywords:** Mortality, Material Deprivation, Metropolitan Area, Urbanization, Small Area, Bayesian Model, Inequalities, Social/Spatial determinants

## Abstract

**Introduction:**

Socioeconomic inequalities affecting health are of major importance in Europe. The literature enhances the role of social determinants of health, such as socioeconomic characteristics and urbanization, to achieve health equity. Yet, there is still much to know, mainly concerning the association between cause-specific mortality and several social determinants, especially in metropolitan areas.

This study aims to describe the geographical pattern of cause-specific mortality in the Lisbon Metropolitan Area (LMA), at small area level (parishes), and analyses the statistical association between mortality risk and health determinants (material deprivation and urbanization level). Fourteen causes have been selected, representing almost 60 % of total mortality between 1995 and 2008, particularly those associated with urbanization and material deprivation.

**Methods:**

A cross-sectional ecological study was carried out. Using a hierarchical Bayesian spatial model, we estimated sex–specific smoothed Standardized Mortality Ratios (sSMR) and measured the relative risks (RR), and 95 % credible intervals, for cause-specific mortality relative to 1. urbanization level, 2. material deprivation and 3. material deprivation adjusted by urbanization.

**Results:**

The statistical association between mortality and material deprivation and between mortality and urbanization changes by cause of death and sex. Dementia and MN larynx, trachea, bronchus and lung are the causes of death showing higher relative risk associated with urbanization. Infectious and parasitic diseases, Chronic liver disease and Diabetes are the causes of death presenting higher relative risk associated with material deprivation. Ischemic heart disease was the only cause with a statistical association with both determinants, and MN female breast was the only without any statistical association. Urbanization level reduces the impact of material deprivation for most of the causes of death. Men face a higher impact of material deprivation and urbanization level, than women, in most cause-specific mortality, even when considering the adjusted model.

**Conclusions:**

Our findings explore the specific pattern of fourteen causes of death in LMA and reveals small areas with an excess risk of mortality associated with material deprivation, thereby identifying problematic areas that could potentially benefit from public policies effecting social inequalities.

**Electronic supplementary material:**

The online version of this article (doi:10.1186/s12939-015-0182-y) contains supplementary material, which is available to authorized users.

## Introduction

Health, and the socioeconomic inequalities affecting it, are of major importance in Europe [1], so taking action to reduce health inequalities should be a high priority at all levels of governance [2, 3]. Although Europe has a tradition of studies that analyse the association between material deprivation and increased mortality [4, 5] and other indicators of ill health [6], most of them have analysed individual data at country level [1]. Hence, their results may not be relevant for municipal policymaking [7]. Fewer studies have been able to identify small area level territories within urban areas [8], specifically in metropolitan areas, where interventions can effectively target the structural determinants of health inequalities.

In recent years, area of residence has been recognised as a social determinant of health [9, 10] and, accordingly, the use of spatial analysis of health outcomes and their predictors has increased. Likewise, the development of spatial methods has rapidly improved [11]. By analysing spatial health-related data, researchers were able to identify the association between health determinants and health outcomes at the level of the municipality [12], city [13] and also at small area level [3, 8, 14]. The small area level is considered the best one to avoid the ecological bias component (the Modifiable Areal Unit Problem) created by heterogeneity and to detect geographical patterns in mortality which would not be evident with larger geographical areas [15].

Material deprivation is one of most well-established health determinants [4, 16]: areas with higher socioeconomic deprivation present a higher mortality risk [17]. This association has already been found for Total mortality [8]; Avoidable mortality amenable to healthcare [3]; Diabetes [12]; Infectious diseases [18]; Cancer [19]; Dementia [20]; Suicide [21]; Ischemic heart disease [16]; Cerebrovascular disease [16]; Chronic liver disease [16] and Traffic injuries [22]. According to Testi and Ivaldi [23], who distinguished between material and social forms of deprivation based on Townsend’s approach [5], the material index is the most suitable measure to explain variations in mortality within an urban area.

Today, the rural–urban gradient is also one of the major influential factors in spatial issues [24]. Moreover, urban areas have important health advantages, particularly in the developing world [13, 25]. However, urbanization amplifies the adverse impacts of material deprivation on mortality [26, 27]. As Diez-Roux et al. [28] point out, an important feature of urban areas is the great heterogeneity in socioeconomic circumstances and resources, resulting in enormous inequality in environmental conditions within cities. This means that the consequences of urbanization are not the same for all.

The literature contains several studies that relate urbanization and mortality: higher urbanization has been associated with Ischemic heart disease [29], Infectious disease [30], Chronic liver disease and Cirrhosis [27] and some cancers [31, 32] and lower levels of urbanization have been associated with Suicide [33], Stomach cancer [32], Diabetes [12] and Dementias [34].

Borrell et al. [8] have shown that socioeconomic inequalities in health tend to be more pronounced in more urbanized areas (where disadvantaged and poor populations are concentrated in marginalized neighbourhoods) and that urban areas have certain special characteristics which can influence the population’s health and can be the targets of specific policies. Therefore, given the growth in the urban population, public health challenges must be concentrated in urban areas and policies must be adopted to this context [35].

According to Singh et al. [36], material deprivation and urbanization indices can serve as important surveillance tools for monitoring health inequalities. However the relationship between material deprivation, urban/rural status, and mortality is complex; hence, careful study is required of the way in which urban–rural differences in disease risk are heterogeneous and often context-specific [25]. In fact, material deprivation and urbanization often co-occur in the same places, which mean that it is important to study the mutual influence of these health determinants upon each other.

Some authors have already identified premature mortality inequalities within Lisbon Metropolitan Area (LMA), due to material deprivation [37]. The persistence of poverty, and social and health inequalities in the LMA, despite the general improvement in all health and social indicators [38], proceeds from previous social and political conditions that, at different levels, are also present in other metropolitan areas or cities in European countries; mainly in those that have had delayed industrialization and urbanization, like Portugal. Thus, particular attention should be given to the consequences of material deprivation on urban health at small area level in this region [39].

Studying mortality in small areas, and associating this with material deprivation and with urbanization levels, allows us to identify factors that drive inequalities, and establish how these determinants contribute to inequalities. The information yielded is critical for implementing and tailoring policies to reduce health inequalities and, considering these results, important lessons can be adduced regarding similar contextual factors (urbanization and material deprivation). The results can also be compared internationally with other metropolitan areas with similar characteristics of urbanization and material deprivation.

As far as we know, this paper is the first in Europe to use small-area data to address mortality inequalities associated with material deprivation adjusted to the urbanization level.

The aims of this paper are to describe the geographical mortality pattern in the parishes of the Lisbon Metropolitan Area (LMA) by cause of death, and to analyse the statistical association between mortality and 1. urbanization, 2. material deprivation, and 3. material deprivation adjusted by urbanization, in the period 1995–2008.

### Study area

The LMA is the main metropolitan area of Portugal in which over ¼ of the Portuguese population lives. In accordance with other Southern European cities, Lisbon’s population is steadily ageing, particularly within the city centre: the population aged 65 or over in the LMA increased by 43.7 % (1991: 12.8 %; 2011: 18.4 %, according to the Portuguese National Statistics Office–INE). Nowadays, the older population in the Lisbon municipality accounts for 24 % of total population (INE, 2011).

Geographically, the LMA is divided into two main areas by the River Tagus: the northern and southern banks. The centre is the city of Lisbon, surrounded by a highly urbanized urban ring (in north) and a less urbanized urban ring (in south and northern border). Between the 1970s and 90s, the population in the north urban belt has grown very fast, mainly due to migrants from other Portuguese regions and former African colonies. Yet, this growth has not always been accompanied by public services, infrastructures, land-use mix concerns, etc., with consequences that are important to study, mainly related with social exclusion and health inequities [40].

## Materials and methods

### Design, source of information and indicators

This study follows an ecological design, as defined within the INEQ-CITIES project [8]. The sources of information were mortality registers (aggregated for the period 1995-2008), the 2001 census for population data and socioeconomic indicators, and the 1998 Urban Areas Classification for urbanization data, all from the INE.

The area of analysis was the parish, the lowest administrative level in Portugal. In the LMA there are 207 parishes belonging to 18 municipalities. The parish borders were stable for a long time and during the study period, decreasing the probability of misregistration of death certificates.

Based on an exploratory analysis of sixty causes of death (INEQ-CITIES list), 14 causes of death were selected, representing almost 60 % of total mortality in LMA between 1995 and 2008 (Table 1). The selection was restricted to causes of death previously associated with material deprivation or urbanization level [3, 8, 12, 16, 18–20, 22, 28, 35, 41] and for which numbers can be expected to be large enough to allow small area analysis.

**Table 1:** ICD Codes (for the 9th and 10th Revision) of the causes of death considered in the study

Cause of Death	ICD10	ICD9
Infectious and parasitic disease	A00-B99	001-139
MN stomach	C16	151
MN colon, rectum, anus and anal canal	C18, C19-C21	153, 154
MN larynx, trachea, bronchus and lung	C32-C34	161, 162
MN female breast	C50	174
MN prostate	C61	185
Diabetes mellitus	E10-E14	250
Dementias	F00-F99 excluded F11-F16, F19	290-319 excluded 304, 305 (.2-.9)
Ischemic heart disease	I20-I25	410-414
Cerebrovascular disease	I60-I69	430-434, 436-438
Chronic liver disease	K70, K73, K74	571
Symptoms, signs and abnormal clinical and laboratory findings	R00-R99 excluded R75	780-799
Transport injuries	V01-V99	E800-E049
Suicide and intentional self-harm	X60-X84	E950-E959

Note: To access information about the codes correspondence you can see, for ICD9, http://icd9cm.chrisendres.com/index.php?action=contents, and for ICD10, http://apps.who.int/classifications/icd10/browse/2015/en

The mortality data by cause of death was aggregated for the period 1995–2008 (*N* = 355,363), disaggregated by age (<15; 15–24; 25–44; 45–64; > = 65), sex (total, male, female) and parish (small area) of LMA. For reasons of confidentiality and lack of information, our database includes 97.7 % of total deaths from the selected causes of death in LMA, meaning that 13,319 deaths have not been considered (e.g., age, sex and parish are not mentioned on death certificates). The study population consisted of residents of LMA in 2001, stratified by the same sex and age groups as the mortality data.

To evaluate the social and economic conditions of the area of residence, a material deprivation index was built. This is a composite indicator that takes into account three dimensions: education, employment and housing conditions. The chosen indicators (from the 2001 Census) were: 1. Illiteracy rate (population with more than 10 years that does not know how to write and read); 2. Unemployment rate (unemployment among the population between 14 and 65 years), and 3. Substandard housing rate (houses without toilet). The material deprivation index was constructed in accordance with the method used by Carstairs and Morris [4]. The variables were standardised (using the z-score method) so that each variable exerted the same influence upon the final result. This index, and the indicators used to build it, have been already applied in other studies about material deprivation in LMA [12, 40]. The material deprivation index was analysed in terciles (t1: lowest level of deprivation; t3: highest level of deprivation).

Finally, the Classification of urban areas, produced by INE, was used to determine the urbanization level. This indicator takes into account population density and urban land use to categorise the Portuguese parishes in three groups: 1. Predominantly rural area, 2. Medium urban area and 3. Predominantly urban area. For the last one, the criteria was population density higher than 500 inhabitants/km^2^ and more than half the territory classified as urban. Since the focus is only on the parishes of a metropolitan area, the first two have been aggregated to represent the “less urbanized” parishes and the last one representing the “most urbanized” parishes. This has been analysed as a dichotomic variable.

### Data analysis

The mortality indicator used for this analysis is the Standardized Mortality Ratio (SMR) for total population of LMA. This variable is dependent on population size since its variance is inversely proportional to the expected values. Thus, areas with low population tend to present estimates with a high variance. When analysing aggregated data from small areas, it is important to consider two sources of variability: first, the spatial dependence between geographical areas, which means that neighbouring areas are more likely to have a similar mortality level than distant areas, according to Tobler’s first law of Geography [42]; second, the non-spatial variability (random variation). In order to take into account this variability we used the hierarchical bayesian model proposed by Besag, York and Mollié obtaining smoothed SMR (sSMR) [43]. This method allows us to produce smoothed estimates, minimizing potential bias while still presenting a valid spatial pattern [3, 44].

The sSMR were estimated for each cause of death and sex with the following model:

$$ {O}_i\sim Poisson\left({E}_i{\theta}_i\right) $$

$$ \mathrm{l}og\left({\theta}_i\right) = \alpha + {S}_i + {H}_i $$

where, for each small area *i*, *O*_*i*_ denoted the observed cases of deaths for a particular cause and gender in the small area *i*, *E*_*i*_ was the expected number of deaths (of each cause and gender) in the small area *i and θ*_*i*_, the relative risk for each specific area and specific cause of death. ∝ represents the intercept, *S*_*i*_ the spatial random effects and H_*i*_ the heterogeneous (non-spatial) effects. The expected numbers of deaths in each area were calculated by indirect standardisation, using the population in 2001 (multiplied by the number of years in the study period: 14 years) and taking as reference mortality rates by gender, age (<15; 15–24; 25–44; 45–64; > = 65) and cause of death in the LMA.

Based on sSMR we measured the probability of excess risk (sSMR > 100), which should also be taken into account when evaluating the statistical evidence provided by estimates of sSMR in each small area.

The geographical distribution of sSMR, calculated through Model 1, was represented using maps of septiles: the dark blue areas have the lowest sSMR and the dark brown ones have the highest. The probability of excess risk was represented using five fixed categories: [0–0.1] (lowest probability sSMR > 100), ]0.1-0.2], ]0.2-0.8], ]0.8-0.9] and ]0.9-1.0] (highest probability sSMR > 100).

The statistical association with the contextual-level variables (material deprivation and urbanization) has been obtained through the application of an ecological regression model that introduces those indicators as explanatory variables.

To evaluate the statistical association between mortality by cause of death and urbanization (Xi) (a dichotomous variable), the regression was formulated as follows:

$$ \log \left({\uptheta}_{\mathrm{i}}\right) = {\upbeta}_1 + {\upbeta}_2{\mathrm{X}}_{\mathrm{i}} + {\mathrm{S}}_{\mathrm{i}} + {\mathrm{H}}_{\mathrm{i}} $$

Where exp(*β*_*2*_) denotes the relative risk of mortality in the more urbanized areas with respect to the less urbanized.

To analyse the relationship between mortality and material deprivation (D_*i*_), we applied a similar model in which material deprivation terciles were introduced as dummy variables:

*D*_2*i*_ = 1 if the small area *i* is in the second tercile group.

*D*_2*i*_ = 0 otherwise

*D*_3*i*_ = 1 if the small area *i* is in the third tercile group.

*D*_3*i*_ = 0 otherwise

For this model, called “based”, the regression was formulated as follows:

$$ \log \left({\theta}_i\right) = {\beta}_1 + {\beta}_2{D}_{2i}+{\beta}_3{D}_{3i} + {S}_i + {H}_i $$

where exp(*β*_*2*_) (respectively exp(*β*_*3*_)) denotes the relative risk of mortality in the areas included in the second tercile (respectively third tercile) group with respect to the included in the first tercile deprivation group.

Finally, we estimated the statistical association between material deprivation and mortality adjusted by urbanization level. For this model, called “adjusted”, the regression was formulated as follows:

$$ \log \left({\theta}_i\right) = {\beta}_1 + {\beta}_2{D}_{2i}+{\beta}_3{D}_{3i}+{\beta}_4{X}_i + {S}_i + {H}_i $$

Where exp(*β*_*2*_) (respectively exp(*β*_*3*_)) are adjusted by urbanization level (dichotomic variable) and denotes the relative risk of mortality in the areas included in the second tercile (respectively third tercile) group with respect to those included in the first tercile deprivation group.

For these three models, the relative risk (RR) estimates were obtained based on their posterior means, along with the corresponding 95 % credible intervals (95%CI). A RR was considered significantly higher or lower than 1 if its 95%CI did not include 1. The posterior distributions were obtained with the “Integrated nested Laplace approximation” (INLA) method.

For all models, an intrinsic conditional autoregressive prior distribution (ICAR) was assigned to the spatial effect, which assumes that the expected value of each area coincides with the mean of the spatial effect of the adjacent areas and has variance of *σ*_*s*_^2^, while the heterogeneous effect is represented using independent normal distributions with mean 0 and variance *σ*_*h*_^2^ [43]. A half-normal distribution with mean 0 and precision 0.0001 was assigned to the standard deviations *σ*_*s*_ and *σ*_*h*_. A vague prior distribution was assigned to the parameters β_1,_ β_2_ and β_3_ [45].

These models were developed using the INLA library (version 3.0.1) and the R statistical package (version R.2.15.2) [46].

## Results

**Fig 1:**
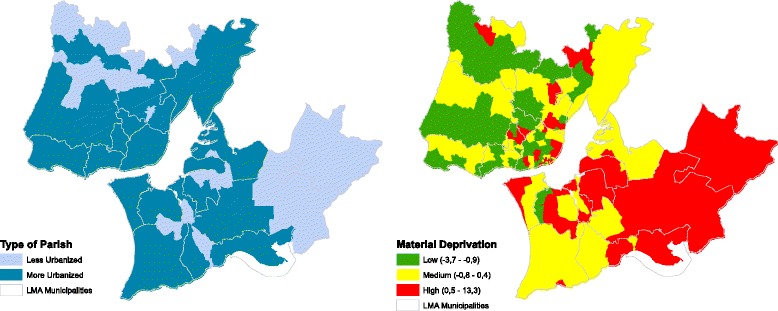
Geographic distribution of the urbanization level (based on Classification of urban areas, 1998) and material deprivation (2001) (green: lower material deprivation; red: higher material deprivation)

The LMA has small areas with different levels of urbanization, material deprivation and mortality (Table 2 and Fig. 1). The geography of material deprivation reveals high levels in the southern river bank and in some parishes of the city centre and periurban areas (in red), and low material deprivation in the west and north of LMA (in green). The most urbanized parishes are in the northern bank. The statistical association between urbanization and material deprivation (chi-square test) was not found.

**Table 2:** Descriptive analysis of the data of the study area: quartile distribution of the number of inhabitants and deaths (by the 14 selected causes) by sex and level of urbanization, 2001

			Less Urbanized Parishes	Most Urbanized Parishes	Total
Number of areas			35	172	207
Population (2001)	Total	P25	10,015	151,381	161,396
		P50	16,697	377,384	394,081
		P75	32,385	638,238	670,623
		Total	88,725	2,573,125	2,661,850
	Men	P25	4900	7,1395	76,295
		P50	8233	179,148	187,381
		P75	16,043	304,270	320,313
		Total	43,861	1,231,798	1,275,659
	Women	P25	5115	79,975	85,090
		P50	8464	197,534	205,998
		P75	16,342	333,816	350,158
		Total	44,864	1,341,327	1,386,191
Mortality (1995–2008)	Total	P25	538	25,344	16,579
		P50	1901	52,074	49,504
		P75	3989	90,902	90,837
		Total	11,913	343,450	355,363
	Men	P25	174	13,369	8483
		P50	920	26,938	25,650
		P75	2155	47,109	47,129
		Total	6275	177,680	183,955
	Women	P25	364	11,975	8028
		P50	981	25,136	23,500
		P75	1834	43,793	43,548
		Total	5638	165,770	171,408

Source: based on Portuguese National Statistics Institute

Table 3 presents the number of deaths and crude mortality rate by cause of death and sex. Cerebrovascular disease and Ischemic heart disease are the most common causes of death in both sexes (15.9 % and 12.6 % of total deaths in LMA, respectively): among men, is Ischemic heart disease; among women is Cerebrovascular disease. In the majority of causes of death, the number is higher in men, but for Diabetes mellitus, Dementia and Cerebrovascular disease, women have higher values.

**Table 3:** Descriptive analysis: total number of Deaths and crude death rates by sex and cause of death in Lisbon Metropolitan Area (aggregated period, 1995–2008)

Causes of Death	Number of Deaths^a^			Crude Rates (total population, *per* 1000)		
	Total	Men	Women	Total	Men	Women
Infectious and parasitic disease	13,533	9665	3868	5.1	7.6	2.8
Malignant neoplasm (MN) stomach	7696	4713	2983	2.9	3.7	2.2
MN colon, rectum, anus and anal canal	12,530	7008	5522	4.7	5.5	4.0
MN larynx, trachea, bronchus and lung	13,550	11,169	2381	5.1	8.8	1.7
MN female breast	6710		6710	2.5	0.0	4.8
MN prostate	5643	5643		2.1	4.4	0.0
Diabetes mellitus	12,723	5525	7198	4.8	4.3	5.2
Dementias	1787	688	1099	0.7	0.5	0.8
Ischemic heart disease	45,392	23,552	21,840	17.1	18.5	15.8
Cerebrovascular disease	57,347	23,437	33,910	21.5	18.4	24.5
Chronic liver disease	5271	4149	1122	2.0	3.3	0.8
Symptoms, signs and abnormal clinical and laboratory findings	20,590	10,295	10,295	7.7	8.1	7.4
Transport injuries	6022	4486	1536	2.3	3.5	1.1
Suicide and intentional self-harm	3267	2447	820	1.2	1.9	0.6
Total of the 14 causes of death	212,113	112,829	99,284	79.7	88.4	71.6
Total Deaths	355,363	183,955	171,408	133.5	144.2	123.7

^a^The deaths where sex, age or parish of residence are missing have not been quantified

Source: based on Portuguese National Statistics Institute, 1995–2008

**Fig. 2:**
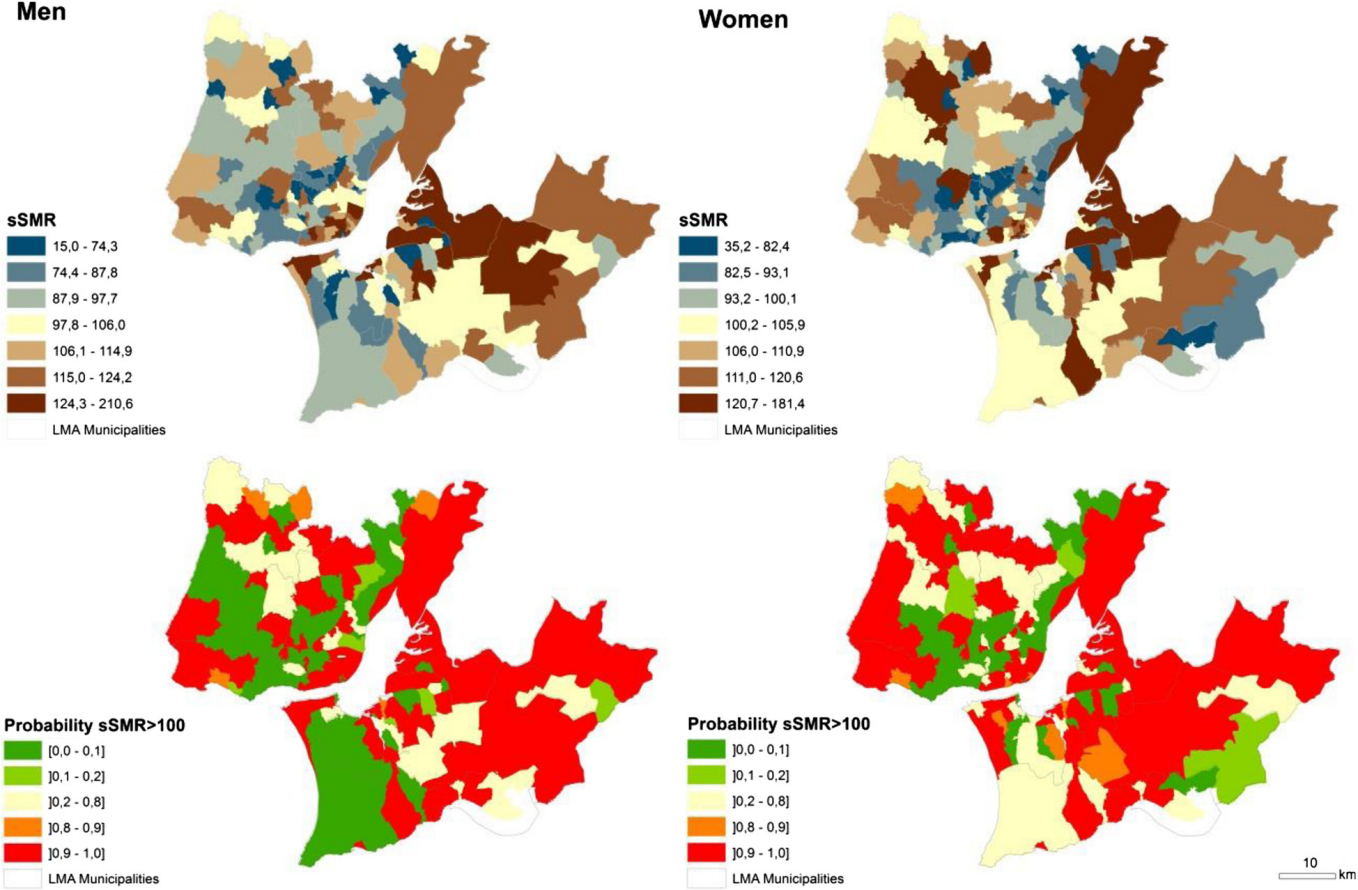
Geographic distribution of the smoothed Standardized Mortality Ratios (sSMR) for the total of the 14 selected causes of death in LMA (blue: low sSMR; brown: high sSMR) and the probability that the sSMR is higher than 100 (green: low risk; red: high risk). Note: the maps for each cause of death are present in the Additional file

The geographical distribution shows that the highest sSMR due to the total selected causes of death, for both genders, are found in some urban areas, located especially in Lisbon city centre. From here, excess mortality risk continues southwards to the southern river bank. The highest deficit in mortality risk for both genders is evident in the areas surrounding the Lisbon city centre, where low mortality risk is concentrated (Fig. 2).

Most of the causes of death follow this centre (highly urbanized small areas) to periphery (lower urbanized small areas) configuration. This is the case for Infectious and parasitic disease; MN of the colon, rectal, anus and anal canal; MN larynx, trachea, bronchus and lung; MN of the female breast; Chronic liver disease and Dementia. In the opposite direction we found Transport injuries and, for men, MN of the stomach and Suicide and intentional self-harm. Additionally, some causes of death present a North/South pattern: MN stomach; Diabetes mellitus and Symptoms, signs and abnormal clinical and laboratory findings show a southern river bank with high risk of mortality (see Additional file 1 ).

Table 4 shows the results of the ecological regression that identified the statistical association between the urbanization and cause-specific mortality: the more urbanized areas present a higher risk (1.35–95 % CI: 1.18-1.53) than the less urbanized ones, especially for men (1.50–95 % CI: 1.26-1.80). The exception occurs in the case of Suicide and intentional self-harm and Transport injuries for men (Table 4). Dementia is the cause of death that presents higher mortality risk for the population living in more urbanized areas (1.94–95 % CI: 1.20-3.01), especially for men (2.31–95 % CI: 1.19-4.30). Although the statistical association in most causes of death is similar between sex, there are some cases where it is only significant for men. That is the case for MN larynx, trachea, bronchus and lung; Infectious and parasitic disease, Diabetes mellitus and Transport injuries.

**Table 4:** Relative Risk (RR) and 95 % credible intervals between urbanization (less urbanized parishes compared with more urbanized parishes) and mortality by cause of death in the Lisbon Metropolitan Area (1995–2008)

Causes of Death	Total			Men			Women		
	RR	2.5 %	97.5 %	RR	2.5 %	97.5 %	RR	2.5 %	97.5 %
Infectious and parasitic disease	1.46	1.15	1.83	1.47	1.12	1.90	1.28	0.96	1.68
MN stomach	0.90	0.76	1.06	0.93	0.77	1.13	0.83	0.67	1.02
MN colon, rectum, anus and anal canal	1.30	1.14	1.50	1.28	1.09	1.50	1.33	1.09	1.62
MN larynx, trachea, bronchus and lung	1.56	1.32	1.83	1.59	1.33	1.89	1.37	0.99	1.88
MN female breast	1.19	0.99	1.43	NA	NA	NA	1.19	0.99	1.43
MN prostate	1.24	1.03	1.50	1.24	1.03	1.50	NA	NA	NA
Diabetes mellitus	1.25	1.07	1.45	1.38	1.13	1.67	1.12	0.93	1.33
Dementias	1.94	1.20	3.01	2.31	1.19	4.30	1.86	1.02	3.23
Ischemic heart disease	1.31	1.16	1.46	1.40	1.23	1.60	1.16	1.01	1.32
Cerebrovascular disease	1.13	1.00	1.28	1.16	0.99	1.34	1.03	0.91	1.16
Chronic liver disease	1.22	0.96	1.55	1.19	0.92	1.53	1.18	0.77	1.77
Symptoms, signs and abnormal findings	1.09	0.93	1.27	1.10	0.92	1.31	1.05	0.87	1.25
Transport injuries	0.86	0.71	1.03	0.81	0.67	0.99	0.88	0.64	1.19
Suicide and intentional self-harm	0.68	0.54	0.84	0.67	0.52	0.85	0.57	0.40	0.80
Total	1.35	1.18	1.53	1.50	1.26	1.80	1.18	1.06	1.31

NA = Not applicable

Table 5 presents the results of the ecological regression that identified the statistical association between the index of material deprivation, in terciles, and cause-specific mortality before and after adjustment by urbanization level. Most causes of death show significant association between cause-specific mortality and material deprivation, mainly for total deaths and for men: more deprived areas have a higher risk than the less deprived ones. The results of both models are very similar: Infectious and parasitic disease; Chronic liver disease; Diabetes mellitus and MN stomach are the main causes of death associated with deprivation, disregarding sex. Yet, according to the base model, people that live in the most deprived tercile have an 18 % higher risk (95 % IC: 1.06-1.32) of dying from one of the fourteen selected causes than the population living in the lowest deprived tercile. With the adjusted model the relative risk is 21 % higher (95 % IC: 1.09-1.35). This reveals that urbanization reduces the effect of material deprivation on mortality in 3 %. For men the figure is 5 %.

**Table 5:** Relative Risk (RR) and 95 % credible intervals between material deprivation (the 2nd and 3^rd^tercile (most deprived) compared with the 1^st^tercile (less deprived)) and mortality by cause of death in Lisbon Metropolitan Area (1995–2008)

Cause of Death		Based model									Adjusted by urbanization level								
	Tercile	Total			Men			Women			Total			Men			Women		
		RR	95 % CI		RR	95 % CI		RR	95 % CI		RR	95 % CI		RR	95 % CI		RR	95 % CI	
Infectious and parasitic disease	T2	1.33	1.18	1.52	1.39	1.21	1.59	1.23	1.08	1.4	1.37	1.20	1.55	1.42	1.23	1.62	1.24	1.08	1.42
	T3	1.74	1.52	2.01	1.81	1.55	2.11	1.58	1.37	1.83	1.80	1.57	2.08	1.87	1.61	2.19	1.60	1.38	1.85
MN stomach	T2	1.18	1.08	1.30	1.19	1.07	1.33	1.18	1.04	1.32	1.18	1.07	1.30	1.19	1.07	1.33	1.17	1.04	1.31
	T3	1.29	1.17	1.44	1.32	1.17	1.49	1.26	1.11	1.43	1.29	1.17	1.43	1.32	1.17	1.49	1.25	1.11	1.42
MN colon, rectum, anus and anal canal	T2	1.02	0.95	1.10	1.09	1.00	1.18	0.95	0.86	1.04	1.03	0.95	1.10	1.09	1.01	1.19	0.95	0.87	1.05
	T3	0.99	0.92	1.08	1.03	0.94	1.13	0.98	0.89	1.09	1.01	0.93	1.10	1.04	0.95	1.14	0.99	0.90	1.10
MN larynx, trachea, bronchus and lung	T2	1.05	0.96	1.14	1.09	0.99	1.19	0.92	0.80	1.05	1.06	0.97	1.15	1.10	1.00	1.20	0.90	0.79	1.03
	T3	1.12	1.02	1.24	1.17	1.06	1.30	0.92	0.79	1.08	1.15	1.05	1.28	1.21	1.10	1.34	0.92	0.79	1.07
MN female breast	T2	1.01	0.92	1.11	NA	NA	NA	1.01	0.92	1.11	1.01	0.92	1.11	NA	NA	NA	1.01	0.92	1.11
	T3	1.03	0.93	1.15	NA	NA	NA	1.03	0.93	1.15	1.04	0.94	1.16	NA	NA	NA	1.04	0.94	1.16
MN prostate	T2	1.05	0.95	1.16	1.05	0.95	1.16	NA	NA	NA	1.06	0.96	1.17	1.06	0.96	1.17	NA	NA	NA
	T3	1.02	0.91	1.14	1.02	0.92	1.14	NA	NA	NA	1.03	0.93	1.16	1.03	0.93	1.16	NA	NA	NA
Diabetes mellitus	T2	1.18	1.09	1.28	1.18	1.07	1.30	1.20	1.08	1.32	1.19	1.09	1.29	1.19	1.07	1.31	1.20	1.09	1.32
	T3	1.30	1.19	1.42	1.25	1.12	1.39	1.35	1.22	1.51	1.31	1.20	1.44	1.26	1.14	1.42	1.36	1.23	1.52
Dementias	T2	1.10	0.84	1.40	1.16	0.83	1.58	1.12	0.81	1.51	1.11	0.85	1.42	1.18	0.84	1.61	1.13	0.82	1.53
	T3	1.10	0.84	1.45	1.23	0.86	1.73	1.05	0.75	1.46	1.14	0.87	1.50	1.29	0.91	1.82	1.08	0.77	1.50
Ischemic heart disease	T2	1.10	1.03	1.18	1.07	0.99	1.16	1.13	1.05	1.22	1.12	1.04	1.34	1.09	1.01	1.17	1.14	1.06	1.22
	T3	1.10	1.01	1.19	1.09	1.00	1.20	1.11	1.03	1.21	1.12	1.04	1.22	1.12	1.03	1.22	1.13	1.04	1.23
Cerebrovascular disease	T2	1.13	1.04	1.22	1.14	1.04	1.25	1.11	1.02	1.19	1.13	1.04	1.23	1.15	1.04	1.26	1.11	1.02	1.20
	T3	1.18	1.08	1.29	1.27	1.15	1.41	1.11	1.02	1.22	1.19	1.09	1.31	1.29	1.16	1.43	1.12	1.02	1.22
Chronic liver disease	T2	1.28	1.12	1.45	1.26	1.10	1.45	1.38	1.13	1.66	1.28	1.13	1.46	1.27	1.11	1.45	1.38	1.14	1.66
	T3	1.53	1.33	1.76	1.50	1.29	1.75	1.71	1.41	2.09	1.55	1.34	1.79	1.52	1.31	1.77	1.73	1.42	2.11
Symptoms, signs and abnormal clinical (…)	T2	1.08	0.97	1.20	1.14	1.01	1.28	1.02	0.90	1.16	1.08	0.97	1.21	1.14	1.01	1.28	1.03	0.90	1.16
	T3	1.15	1.02	1.31	1.25	1.10	1.44	1.05	0.91	1.21	1.16	1.03	1.32	1.27	1.11	1.45	1.05	0.91	1.21
Transport injuries	T2	1.24	1.11	1.38	1.29	1.14	1.44	1.16	0.97	1.37	1.23	1.10	1.37	1.28	1.14	1.43	1.16	0.97	1.37
	T3	1.21	1.06	1.37	1.29	1.13	1.48	1.06	0.88	1.29	1.19	1.06	1.36	1.27	1.12	1.46	1.06	0.87	1.28
Suicide and intentional self-harm	T2	1.14	0.99	1.31	1.18	1.01	1.37	1.05	0.83	1.31	1.13	0.98	1.28	1.17	1.01	1.35	1.02	0.81	1.27
	T3	1.11	0.95	1.30	1.19	1.01	1.42	0.89	0.69	1.15	1.08	0.93	1.26	1.16	0.98	1.37	0.87	0.68	1.12
Total	T2	1.15	1.04	1.27	1.13	0.97	1.31	1.12	1.03	1.21	1.17	1.06	1.28	1.16	1.00	1.33	1.13	1.04	1.21
	T3	1.18	1.06	1.32	1.24	1.06	1.46	1.11	1.02	1.21	1.21	1.09	1.35	1.29	1.11	1.51	1.12	1.03	1.23

NA = Not applicable

**Fig. 3:**
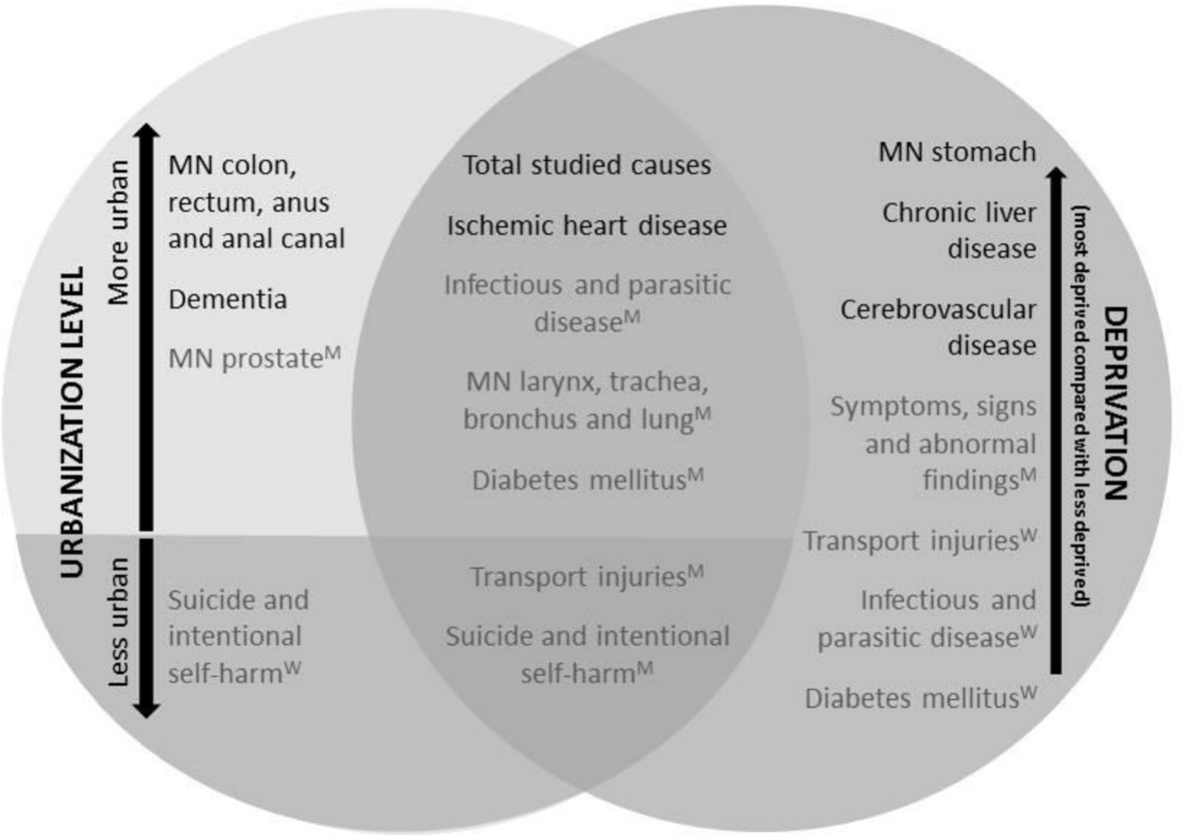
Association between cause-specific mortality by sex and material deprivation (T3: highest material deprivation) and urbanization level. Note: When the associations have only been found for one gender, there is an indication about it: M = only found for men; W = only found for women)

Figure 3 and Table 6 presents the causes of deaths that show statistical association with deprivation and urbanization. The only cause of death presenting a statistical association with both health determinants for men and women is Ischemic heart disease and, in addition, the total studied causes. The statistical association with both health determinants was found for men on Infectious and parasitic disease, MN larynx, trachea, bronchus and lung, Diabetes mellitus, Transport injuries and Suicide and intentional self-harm. The causes of death that do not show any statistical association with material deprivation and urbanization level are MN female breast, MN larynx and Symptoms, signs and abnormal findings, all for women (Table 6).

**Table 6:** Causes of death showing (or not) a significant association with urbanization level and/or material deprivation (most deprived compared with less deprived)

		Without statistical association with Material deprivation	With statistical association with Material deprivation
With statistical association with Urbanization	With statistical association	MN colon, rectum, anus and anal canal	Infectious and parasitic disease^TM^
		MN prostate	MN larynx, trachea, bronchus and lung^TM^
		Dementia	Diabetes mellitus^TM^
		^a^Suicide and intentional self-harm^TW^	Ischemic heart disease
			Cerebrovascular disease^T^
			^a^Transport injuries^M^
			^a^Suicide and intentional self-harm^M^
			Total
	Without statistical association with Urbanization	MN female breast	MN stomach
		MN larynx, trachea, bronchus and lung^W^	Chronic liver disease
		Symptoms, signs and abnormal findings^W^	Symptoms, signs and abnormal findings^TM^
			Transport injuries^TW^
			Infectious and parasitic disease^W^
			Diabetes mellitus^W^
			Cerebrovascular disease^MW^

Notes:^a^ reverse statistical association. ^T^ only for Total.^M^ only for Men ^W^ only for Women

## Discussion

Our results show that: 1. There is a similar geographical pattern of material deprivation and risk of mortality 2. There is a statistical association between mortality and material deprivation, mainly for Infectious and parasitic diseases, Chronic liver disease and Diabetes; 3. There is a statistical association between mortality and urbanization, mainly for Dementia and MN larynx, trachea, bronchus and lung; 4. The urbanization level reduces the impact of material deprivation on mortality for most of the causes; and 5. Socioeconomic inequalities in mortality associated with urbanization level and material deprivation were more pronounced for men than for women.

Firstly, our results indicate that there is substantial intra-urban variation in risk by cause of death, presenting two geographic patterns of mortality across the LMA: city centre versus periphery, and northern river bank versus southern river bank. These two patterns are due to the degree of urbanization, older population rate (particularly in Lisbon city centre) and social and economic contrast between the two river banks: the northern municipalities near the city of Lisbon have experienced a long-term urbanization and suburbanization process, and have the capacity to attract investment and highly-qualified services and human resources; the southern municipalities, on the other hand, have low levels of urbanization and higher unemployment and unqualified workers [47]. The geography of material deprivation also reveals high levels on the southern river bank. The city centre shows high and low levels of material deprivation, which could be one of the reasons for heterogeneity in the city centre (high and low levels of risk of mortality by 14 causes of death). The city centre versus periphery has also been found by other authors in Europe [3, 8]. A similar pattern of intra-urban variability and area effects on mortality, indicating unequal chances of health between different areas, was also revealed by Diez-Roux [28] for Buenos Aires.

Secondly, there is a statistical association between mortality and material deprivation and between mortality and urbanization. The main causes of death in LMA (representing 40.1 % of total mortality) are associated with both material deprivation and/or urbanization level. Nevertheless, some causes show a statistical association with only one health determinant and for one gender. For instance, Ischemic heart disease was the only cause with statistical association with both determinants for both genders.

The association we found between cause-specific mortality and urbanization level confirms the results of other authors [12, 27, 29, 31, 32, 34, 36]. However, we did not find association for MN female breast and Chronic liver disease as other authors [14, 35]. Moreover, in contrast to other authors we found that Dementia, Diabetes mellitus and Stomach cancer have a statistical association with urbanization level. Suicide and intentional self-harm and Transport injuries are the causes of death that show a reverse association with urbanization (higher in less urbanized areas). For Suicide, other authors achieved the same evidence for Portugal [48, 49] and other countries [33]. This may be related with social and economic factors, namely social isolation, stigma towards mental disorders (especially in men) and easy access to highly toxic pesticides [21, 33]. Regarding association between cause specific mortality and material deprivation, as in other studies we also found that mortality increases alongside material deprivation [3, 17, 26, 50, 51]. However, in contrast with other authors, we did not find an association between material deprivation and Dementia. Previous studies related with infectious causes of death (Tuberculosis and AIDS) have already indicated high mortality rates in LMA [52, 53]. Compared with other authors [3, 14, 35] that analysed the association between causes of death and material deprivation in European cities, including the LMA, we also found a clearer association between both. Nevertheless, there are some differences: 1. Mari Dell’Olmo et al. [35] found an inversely significant association between material deprivation and MN female breast, while we did not found any; 2. In our study we did not find an association for Suicide and intentional self-harm for women, as Gotsens et al. [14] and 3. we found a significant association for Ischemic heart disease that Marí-Dell’Olmo et al. [35] only found for women.

Thirdly, urbanization level has the ability to reduce the association of material deprivation with mortality. Some authors argue that urbanization level may be a confounding variable in the association between material deprivation and mortality [26]; others already state (for chronic liver disease and cirrhosis) that the effect is not significant enough to change the association [27]. In our study, although most of the causes of death show a higher (although slighter) relative risk with the adjusted model, especially for men, the causes of death that present a significant statistical association in the based and adjusted model are the same. In the adjusted model, the total causes of death only show a 3 % higher relative risk. For men is 5 %. Infectious and parasitic disease and MN Larynx, trachea, bronchus and lung are the causes of death that reveal higher discrepancy between the based and adjusted models. In fact, as higher the relative risk that we found between mortality and urbanization, the greater the difference between the based and adjusted models. Nevertheless, Dementia was the cause of death showing the highest relative risk for urbanization but in the adjusted model this cause of death continued to present no statistical association with material deprivation. Unlike Dolk et al. [26], we conclude that mortality has a stronger relationship with material deprivation, and the putative excess risk due to urbanization within metropolitan areas is small.

Finally, socioeconomic inequalities in mortality were more pronounced for men than for women with both models, for association with urbanization level and material deprivation. Besides, there are more causes of death with a significant statistical association for men than for women. These sex inequalities have also been described in other studies [3, 8, 22, 27, 35]. In the based model (association between mortality and material deprivation, without adjustment for urbanization) women only have higher statistical association for Diabetes mellitus, Ischemic heart disease and Chronic liver disease. The same occurs with the adjusted model (association between mortality and material deprivation, adjusted for urbanization). Furthermore, the statistical association with both health determinants was only found for Ischemic heart disease and, in men, for Infectious and parasitic disease, MN larynx, trachea, bronchus and lung, Diabetes mellitus, Transport injuries and Suicide and intentional self-harm. This shows us that women are not so influenced by material deprivation and urbanization as men.

As other authors claim [54], people with lower socioeconomic status are more likely to live in metropolitan areas that are more detrimental to health [52, 53]. Improving the socioeconomic determinants of health in those neighbourhoods is crucial to improve the health of the population and to reduce inequalities, because interventions have the greatest potential impact, as stated in the “health impact pyramid” [9, 55]. The conclusions highlight that parishes should be targeted by interventions designed to tackle health inequalities [9]. They also reveal the need for considering the urban territory as a diverse and complex system where health determinants must be analysed through a systematic approach that requires the articulation of mediating mechanisms and analysis of confounding variables [56], such as urbanization level [26].

### Strengths and limitations

As far as we know, this paper is the first one that aimed at measuring and identifying the association between material deprivation, urbanization and mortality within a metropolitan area. Further, it is the pioneer research in Portugal that uses small area data to present mortality inequalities.

However, there are a number of limitations which may impact on the findings presented. First of all, mortality due to Symptoms, signs and abnormal clinical and laboratory findings cause of death is high, representing 5.8 % of total mortality. As a consequence, the other causes here studied may be under-represented, especially Suicide, Diabetes mellitus [57] and Cancer. Second, due to statistical confidentiality, the National Statistics Office only gave access to aggregated mortality data from fourteen years. This time-aggregation was imposed to have access to space-disaggregation data and did not allow to apply time-series cross-sectional analysis. Third, cause-specific mortality maps can only be used to indicate potential problems in material deprivation and urbanization level at small area level, which then have to be studied with more specific information and better local data on the relation between health determinants and health outcomes. The fourth limitation is related with population mobility. As we only have access to the deceased person’s last place of residence, we do not know how long s/he had been living there and how long s/he had been exposed to material deprivation. Furthermore, material deprivation is defined in the period 1995–2008 in the same way as in 2001. However, there were changes over these fourteen years in unemployment and in the number of substandard housing, although the geographical pattern had not changed. Fifth, we were not able to explore if there was an interaction effect between urbanization and material deprivation. Although statistically this could be done, the small number of less urbanized areas does not give us enough sample power to estimate these interactions. Finally, in terms of methodology, there are two main issues: (i) the standardization of mortality data took into account a structure of four age groups, which does not entirely remove the confounding effect of age; and (ii) the existence of statistical associations between the characteristics of place of residence and mortality patterns may be carefully interpreted in terms of causality [58].

### Conclusions and recommendations

Our findings can extend current knowledge by showing spatial patterns of cause-specific mortality in the LMA, identifying small areas with an excess risk of mortality associated with material deprivation and thereby pointing at problematic areas that could potentially benefit from public policies addressing specific causes of death and the effect of social inequalities. These results highlight the need to implement effective policies to reduce inequalities, namely through the intervention of government institutions (local and regional) on specific areas within the metropolitan area [59, 60]. Physical and social environments in neighbourhoods can be overtly hazardous. For instance, evidence about local risk factors (unemployment, illiteracy and poor housing conditions) associated with the Infectious and parasitic diseases, Chronic liver disease and Diabetes mellitus, within LMA, will potentially support the development of local interventions addressing those social and material conditions. Local governments are in a better position to tackle some of these health determinants, by implementing social programmes and built environment interventions aiming to reduce poverty and to improve constructed features that encourage healthy behaviours. Policy measures tackling unemployment and poverty include strategies reducing supply-side unemployment (e.g., education and training schemes, self-employment assistance), the number of families at risk of poverty (e.g., social benefits for low-income individuals and families, local council taxes reduction, affordable housing, access of disadvantaged population groups to health services, lifelong learning actions). Interventions targeting the built environment, urban design and planning in economically disadvantaged small-areas includes various interventions related with physical surroundings (e.g., buildings, green urban spaces, schools, road systems and other infrastructures), housing conditions (e.g., rehousing, refurbishment and community regeneration) and food environment (e.g., increasing the availability of healthy food choices, activities to encourage families to purchase healthier food options) [40].

As so, our results must be transferred to the local stakeholders, especially from sectors such as urban planning, culture, leisure, education, environment, social services and housing, due to their ability to exacerbate or reduce intra-urban health inequalities [61, 62].

## Additional file

Additional file 1: Maps of smoothed Standardized Mortality Ratios(sSMR) for specific cause of death in LMA and the probability that the sSMR is higher than 100 Description of data: Figures shows mortality maps for specific causes of death in the Lisbon Metropolitan Area for men and women separately. The colours represent smoothed Standardized Mortality Ratios (sSMR): the dark blue areas have the lowest sSMR and the dark brown ones have the highest. Next to each map showing the level of mortality for each small area, there is a map giving the probability that the shown sSMRs are above 100. This is the credibility level and represents the Bayesian correspondent to confidence intervals. On this credibility map, red indicates a probability of 90-100 % that an sSMR is higher than 1 and green colour indicates with the same probability that it is lower than 1.

## Abbreviations

ICD9: International Statistical Classification of Diseases and Related Health Problems 9th Revision
ICD10: International Statistical Classification of Diseases and Related Health Problems 10th Revision
INE: Portuguese National Statistics Office
LMA: Lisbon Metropolitan Area
MN: Malignant neoplasm
RR: relative risk
sSMR: smoothed Standardized Mortality Ratio
