# Supplementary material for: Mortality, material deprivation and urbanization: exploring the social patterns of a metropolitan area
Source: Int J Equity Health. 2015 Jun 9;14:55. doi: 10.1186/s12939-015-0182-y (PMC4483227; doi:10.1186/s12939-015-0182-y)
Supplement: Additional file 1: — Maps of smoothed Standardized Mortality Ratios(sSMR) for specific cause of death in LMA and the probability that the sSMR is higher than 100 Description of data: Figures shows mortality maps for specific causes of death in the Lisbon Metropolitan Area for men and women separately. The colours represent smoothed Standardized Mortality Ratios (sSMR): the dark blue areas have the lowest sSMR and the dark brown ones have the highest. Next to each map showing the level of mortality for each small area, there is a map giving the probability that the shown sSMRs are above 100. This is the credibility level and represents the Bayesian correspondent to confidence intervals. On this credibility map, red indicates a probability of 90-100 % that an sSMR is higher than 1 and green colour indicates with the same probability that it is lower than 1. [file 12939_2015_182_MOESM1_ESM.docx]

**Additional file: Maps of Smoothed standardized mortality ratios (sSMR) for specific cause of death in LMA and the probability that the sSMR is higher than 100**

Smoothed standardized mortality ratios (sSMR) for **Infectious and parasitic disease** in LMA and the probability that the sSMR is higher than 100


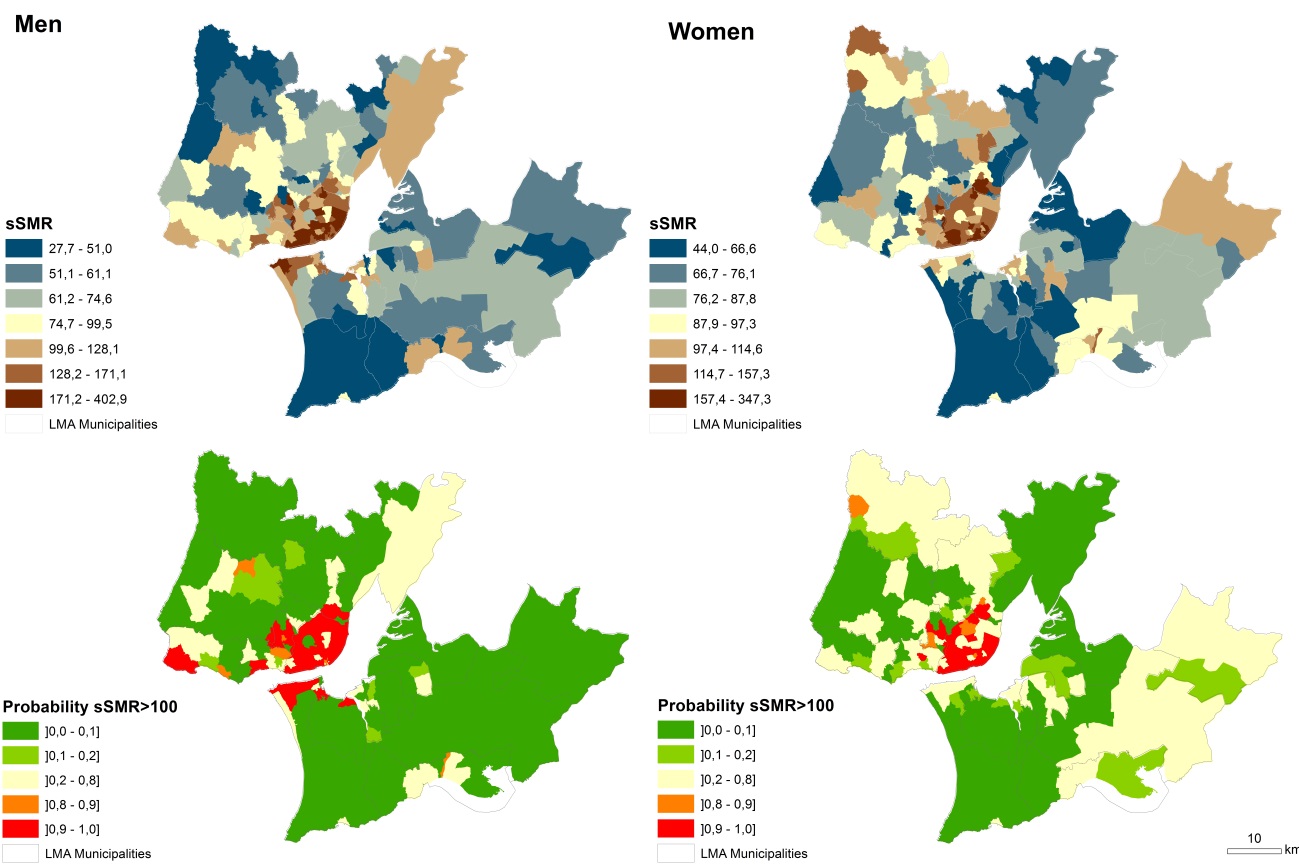


Source: based on Portuguese National Statistics Institute, 1995-2008.

Smoothed standardized mortality ratios (sSMR) for **MN stomach** in LMA and the probability that the sSMR is higher than 100


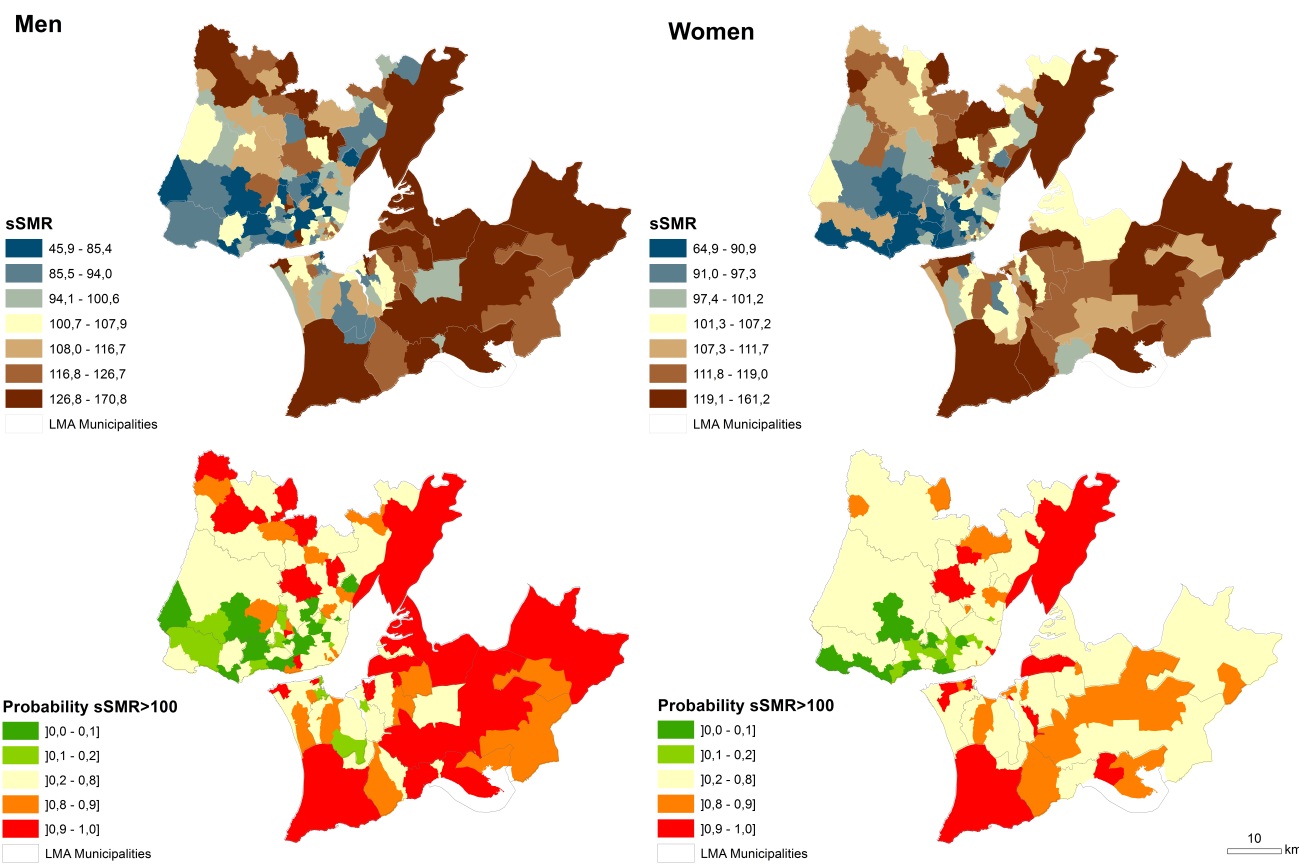


Source: based on Portuguese National Statistics Institute, 1995-2008.

Smoothed standardized mortality ratios (sSMR) for **MN colon, rectum, anus and anal canal** in LMA and the probability that the sSMR is higher than 100


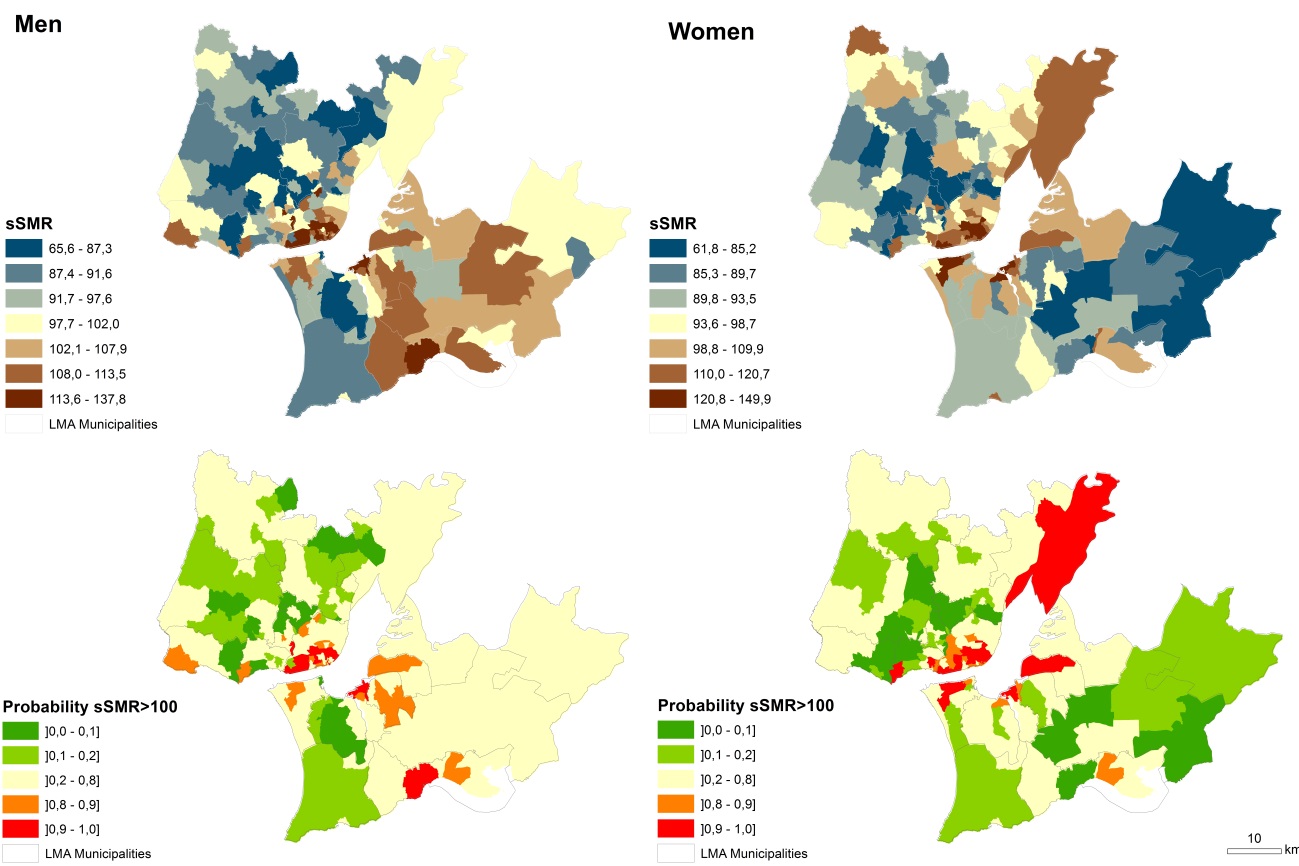


Source: based on Portuguese National Statistics Institute, 1995-2008.

Smoothed standardized mortality ratios (sSMR) for **MN larynx, trachea, bronchus and lung** in LMA and the probability that the sSMR is higher than 100


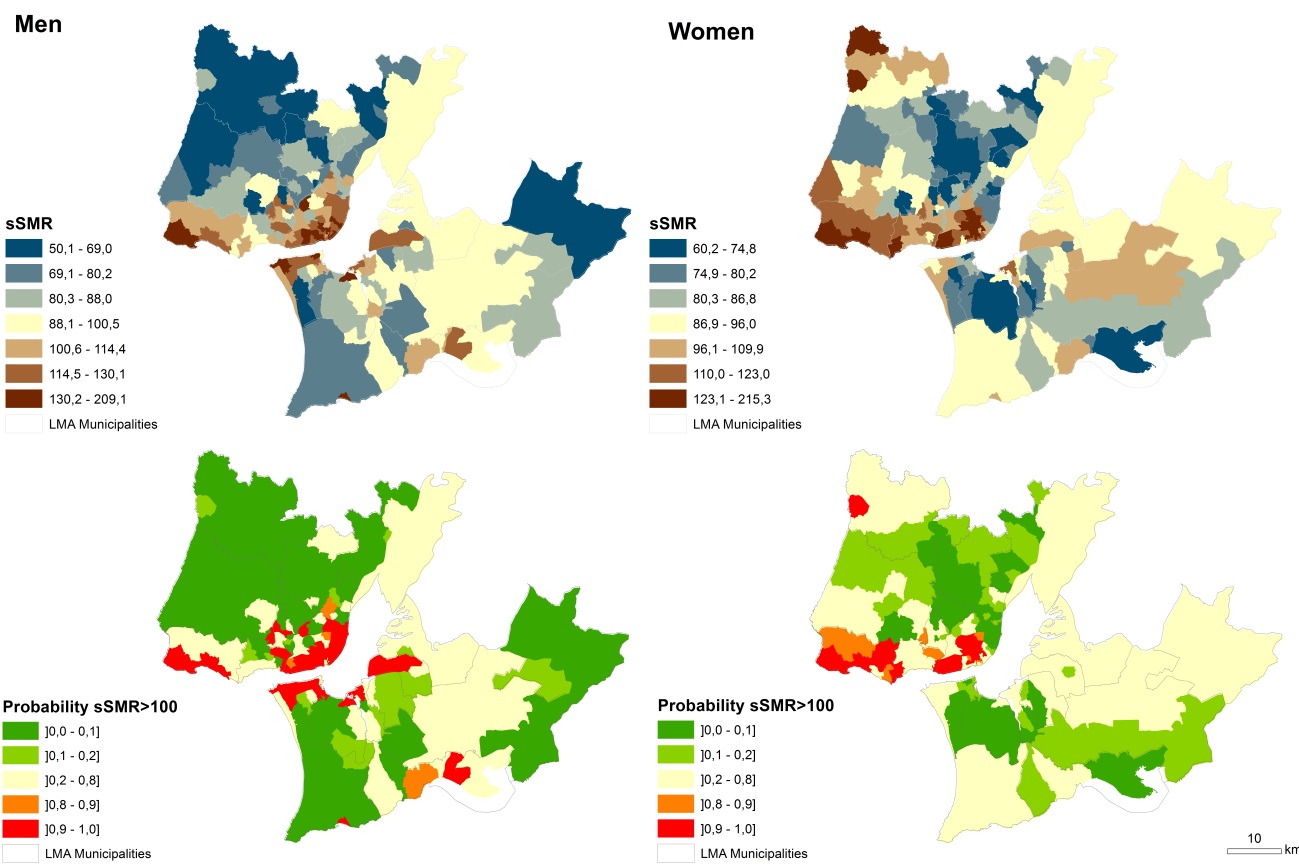


Source: based on Portuguese National Statistics Institute, 1995-2008.

Smoothed standardized mortality ratios (sSMR) for **MN female breast (women) and MN prostate (men)** in LMA and the probability that the sSMR is higher than 100


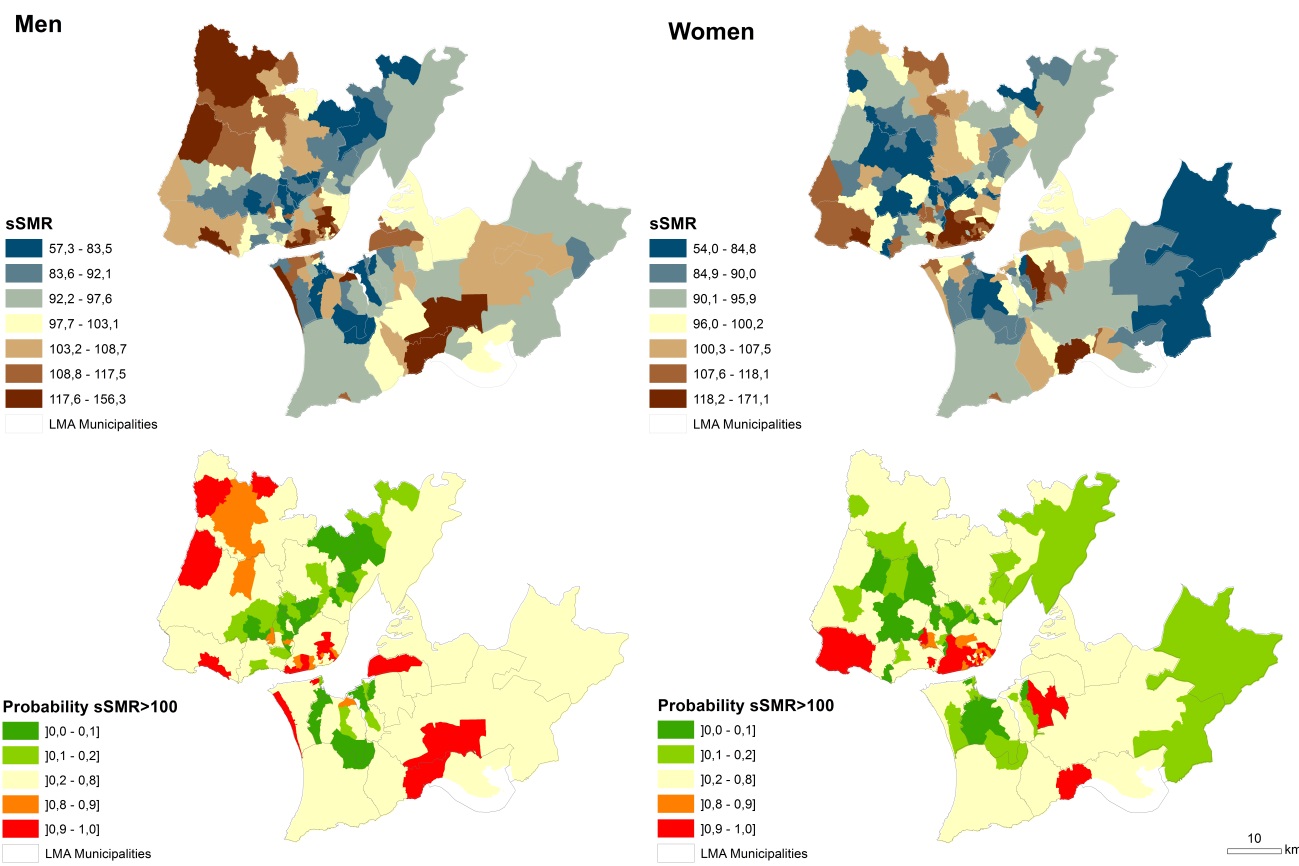


Source: based on Portuguese National Statistics Institute, 1995-2008.

Smoothed standardized mortality ratios (sSMR) for **Diabetes mellitus** in LMA and the probability that the sSMR is higher than 100


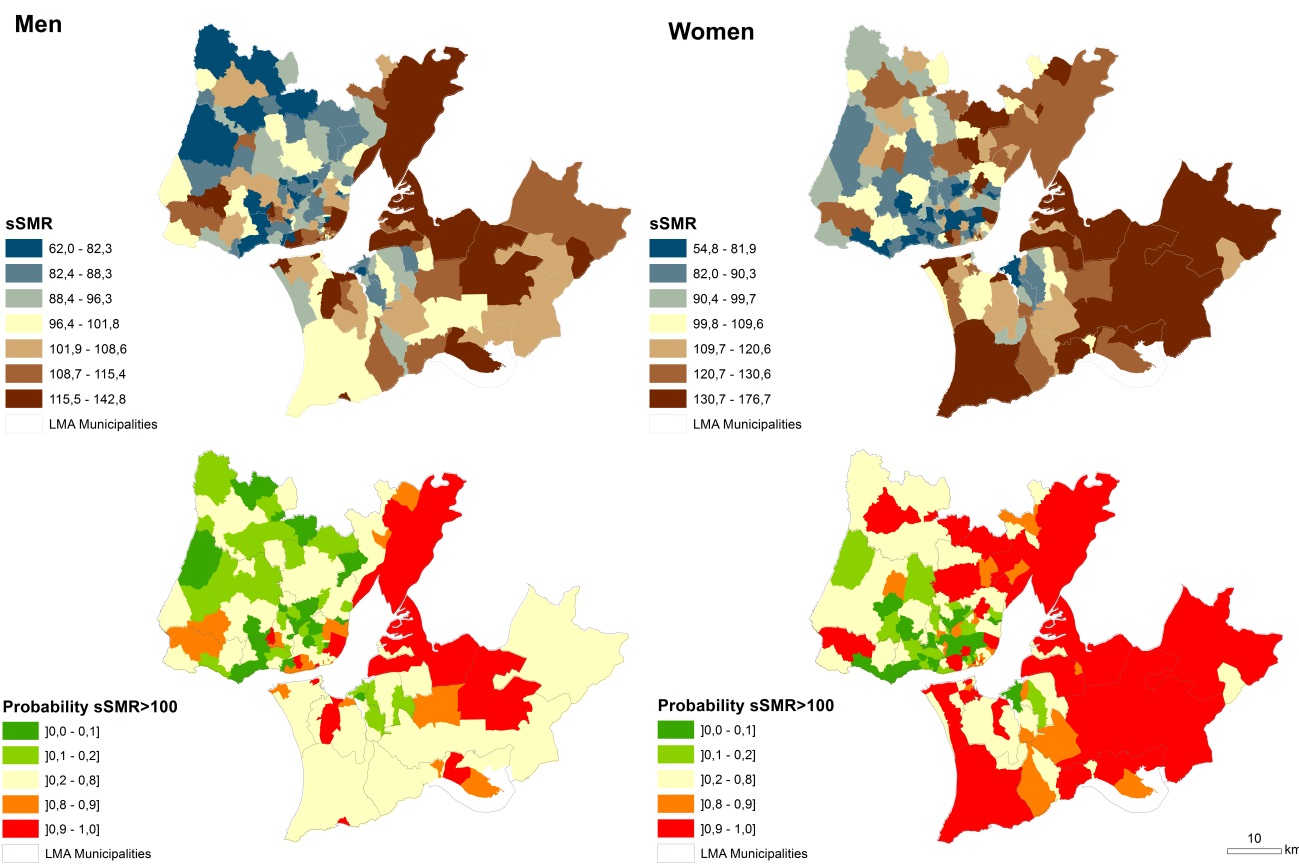


Source: based on Portuguese National Statistics Institute, 1995-2008.

Smoothed standardized mortality ratios (sSMR) for **Dementias** in LMA and the probability that the sSMR is higher than 100


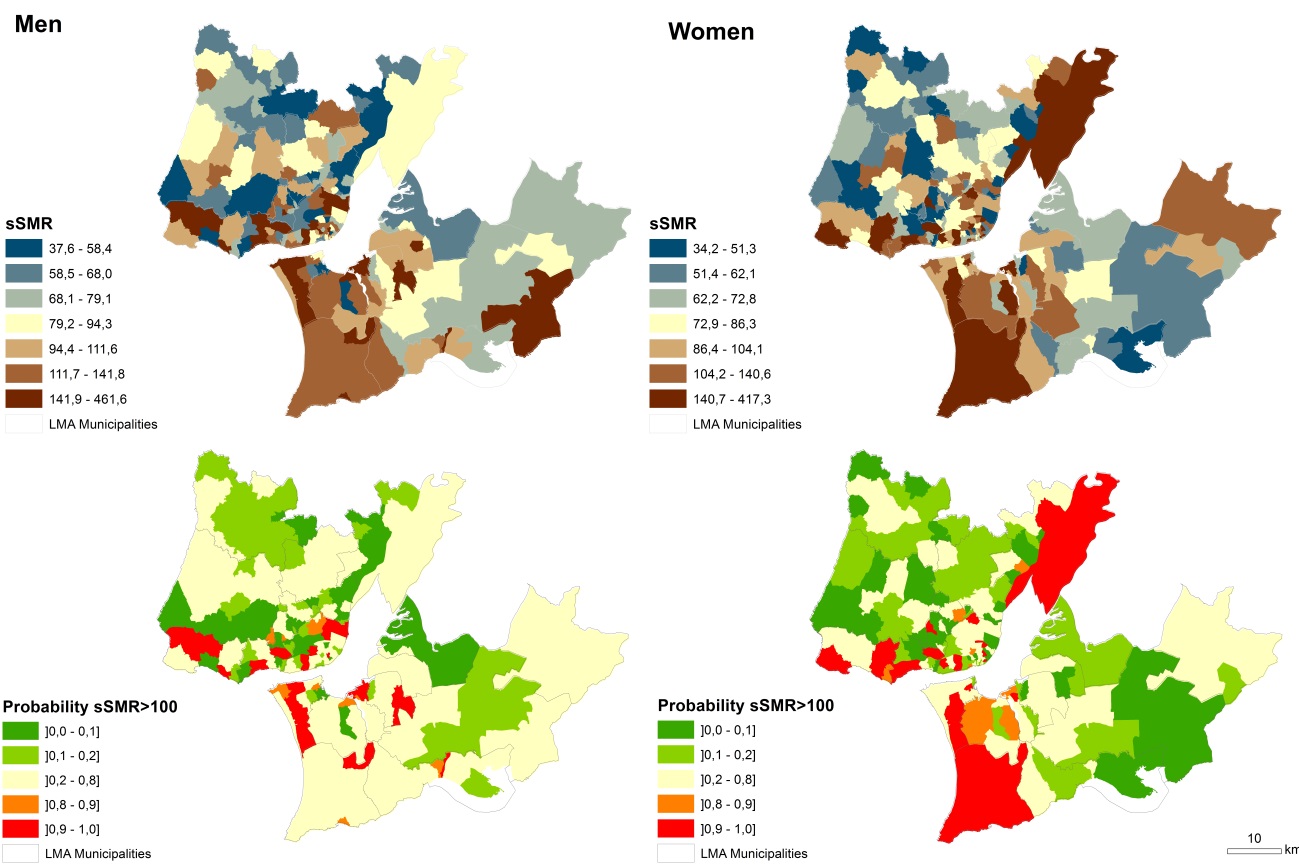


Source: based on Portuguese National Statistics Institute, 1995-2008.

Smoothed standardized mortality ratios (sSMR) for **Ischemic heart disease** in LMA and the probability that the sSMR is higher than 100


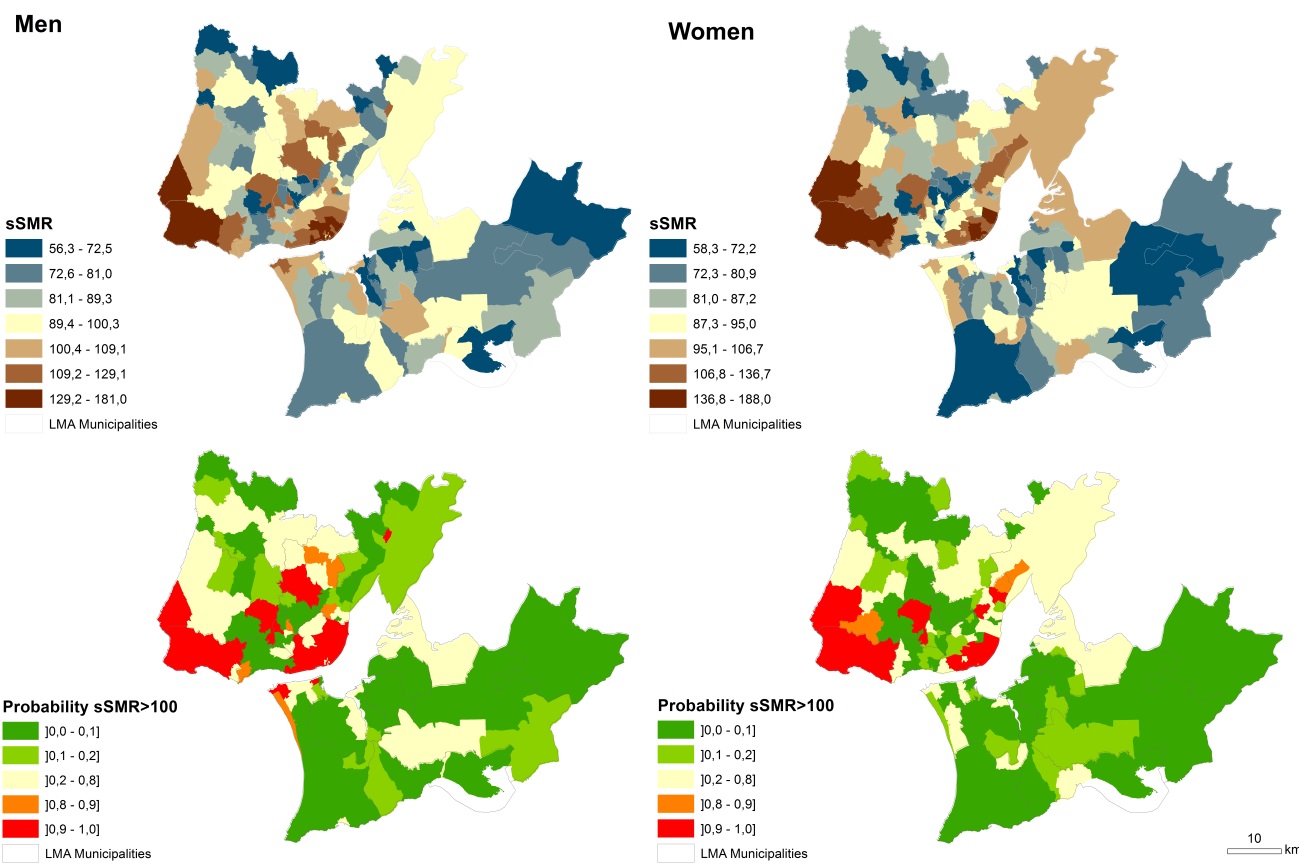


Source: based on Portuguese National Statistics Institute, 1995-2008.

Smoothed standardized mortality ratios (sSMR) for **Cerebrovascular disease** in LMA and the probability that the sSMR is higher than 100


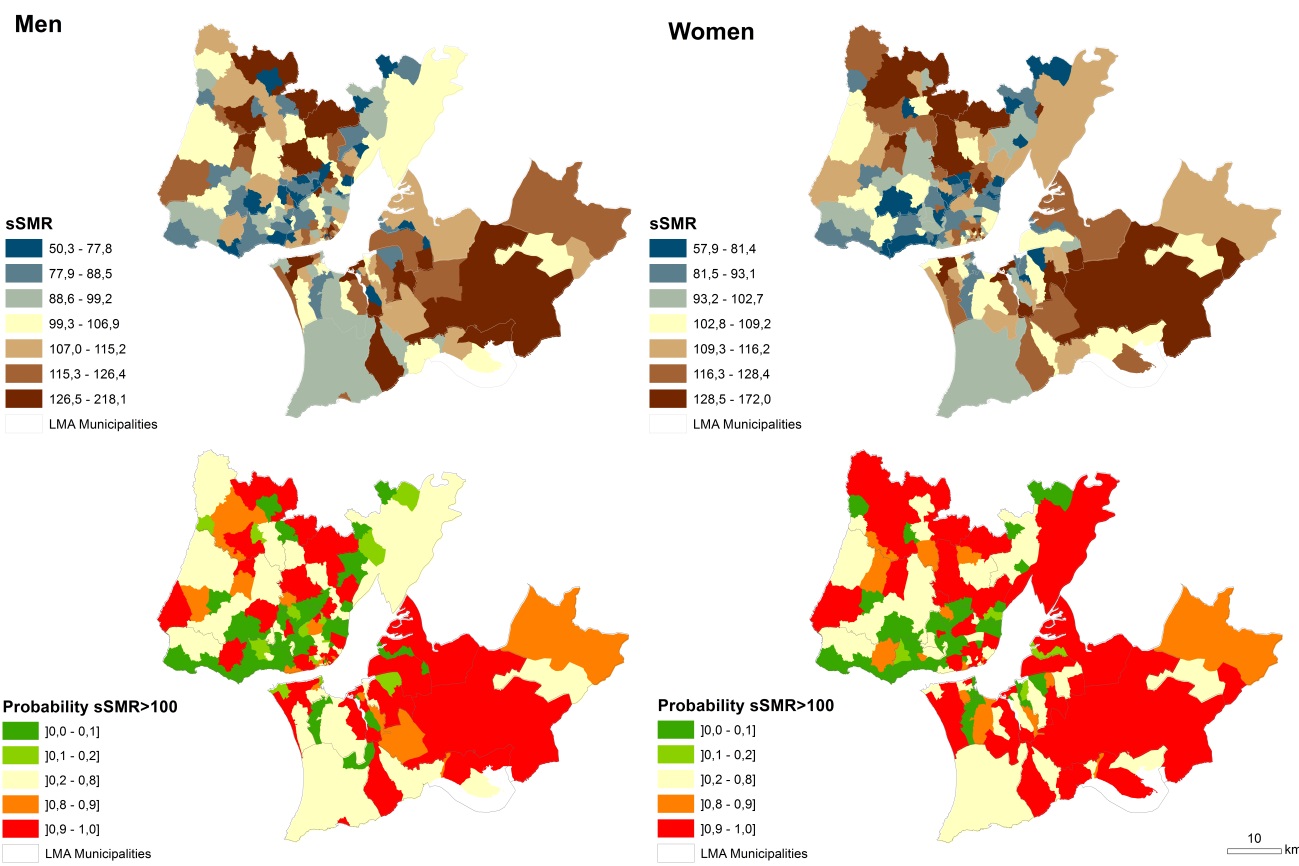


Source: based on Portuguese National Statistics Institute, 1995-2008.

Smoothed standardized mortality ratios (sSMR) for **Chronic liver disease** in LMA and the probability that the sSMR is higher than 100


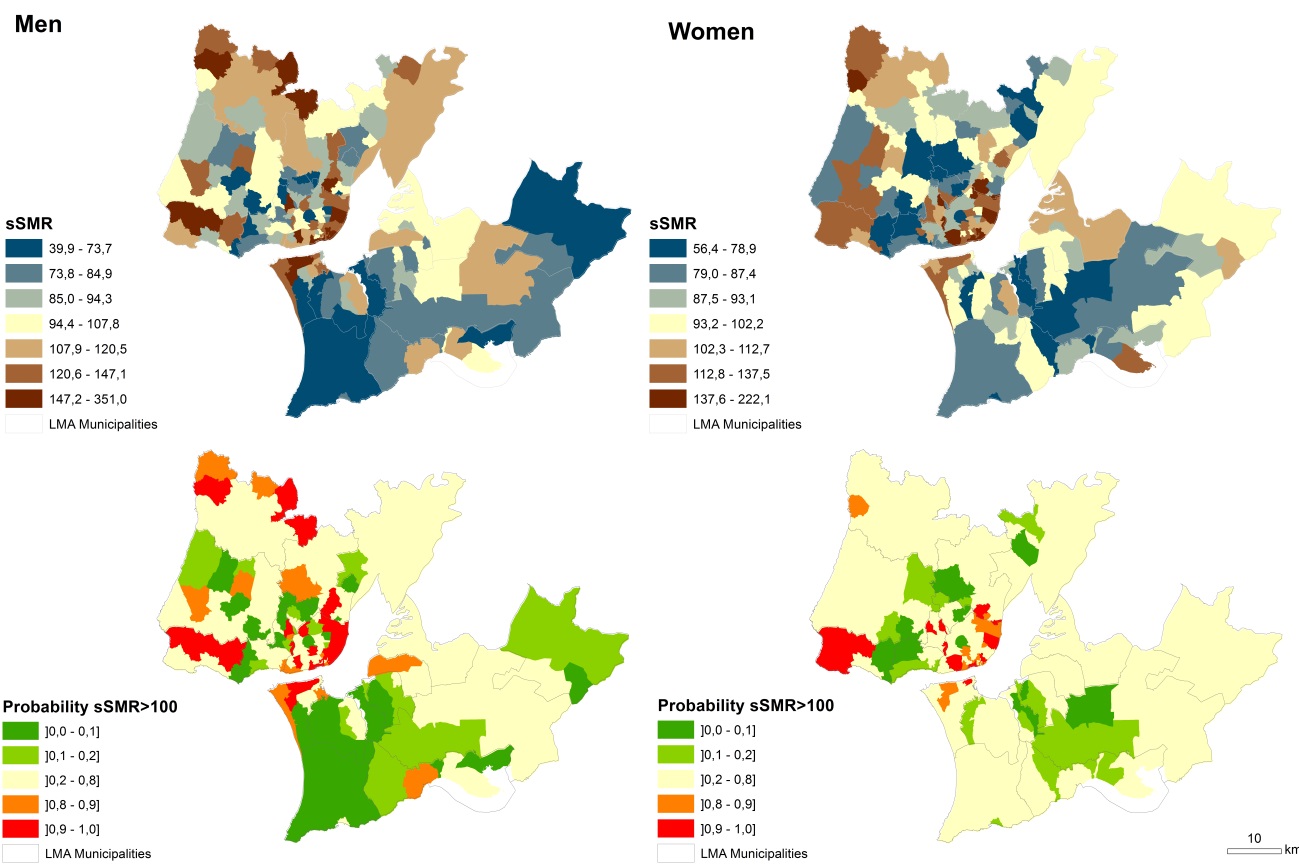


Source: based on Portuguese National Statistics Institute, 1995-2008.

Smoothed standardized mortality ratios (sSMR) for **Symptoms, signs and abnormal clinical and laboratory findings** in LMA and the probability that the sSMR is higher than 100


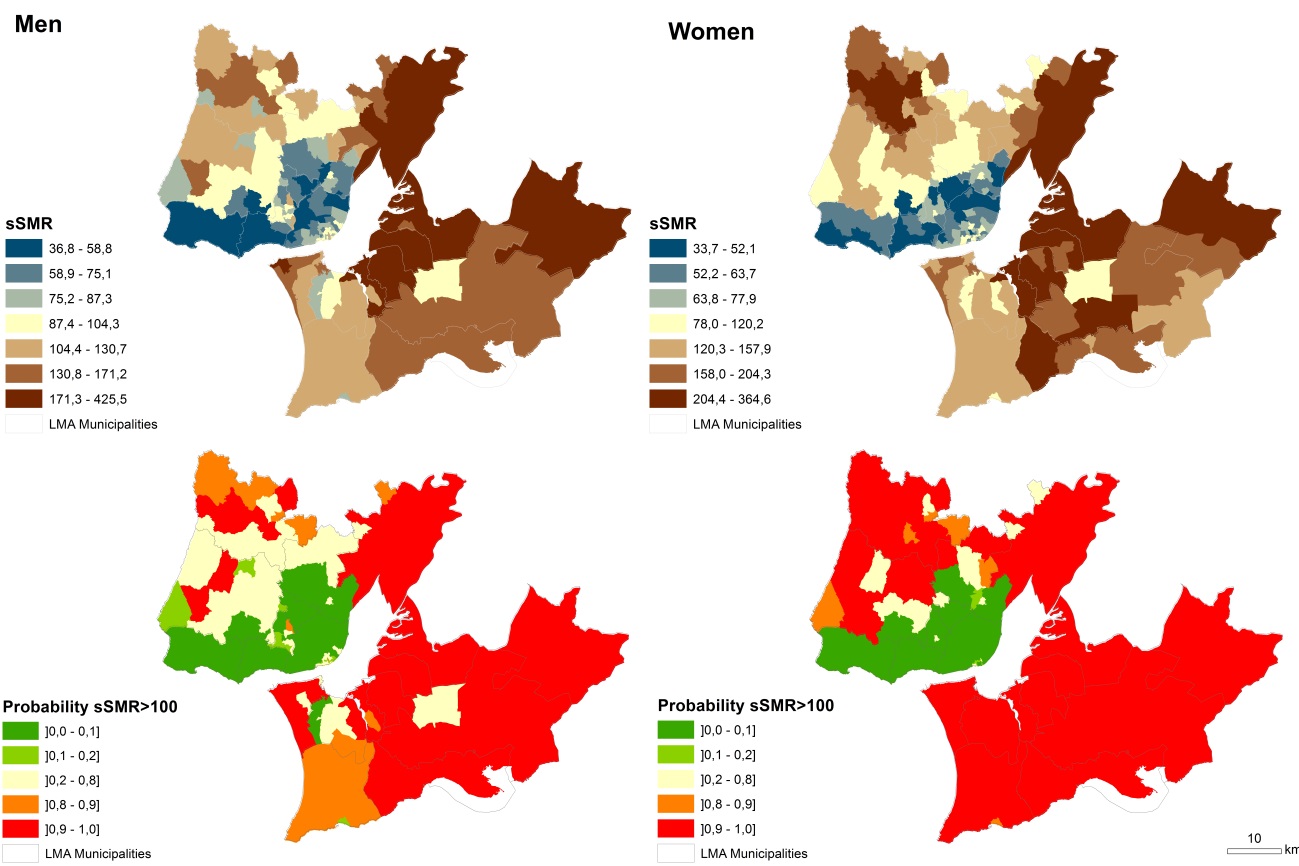


Source: based on Portuguese National Statistics Institute, 1995-2008.

Smoothed standardized mortality ratios (sSMR) for **Transport injuries** in LMA and the probability that the sSMR is higher than 100


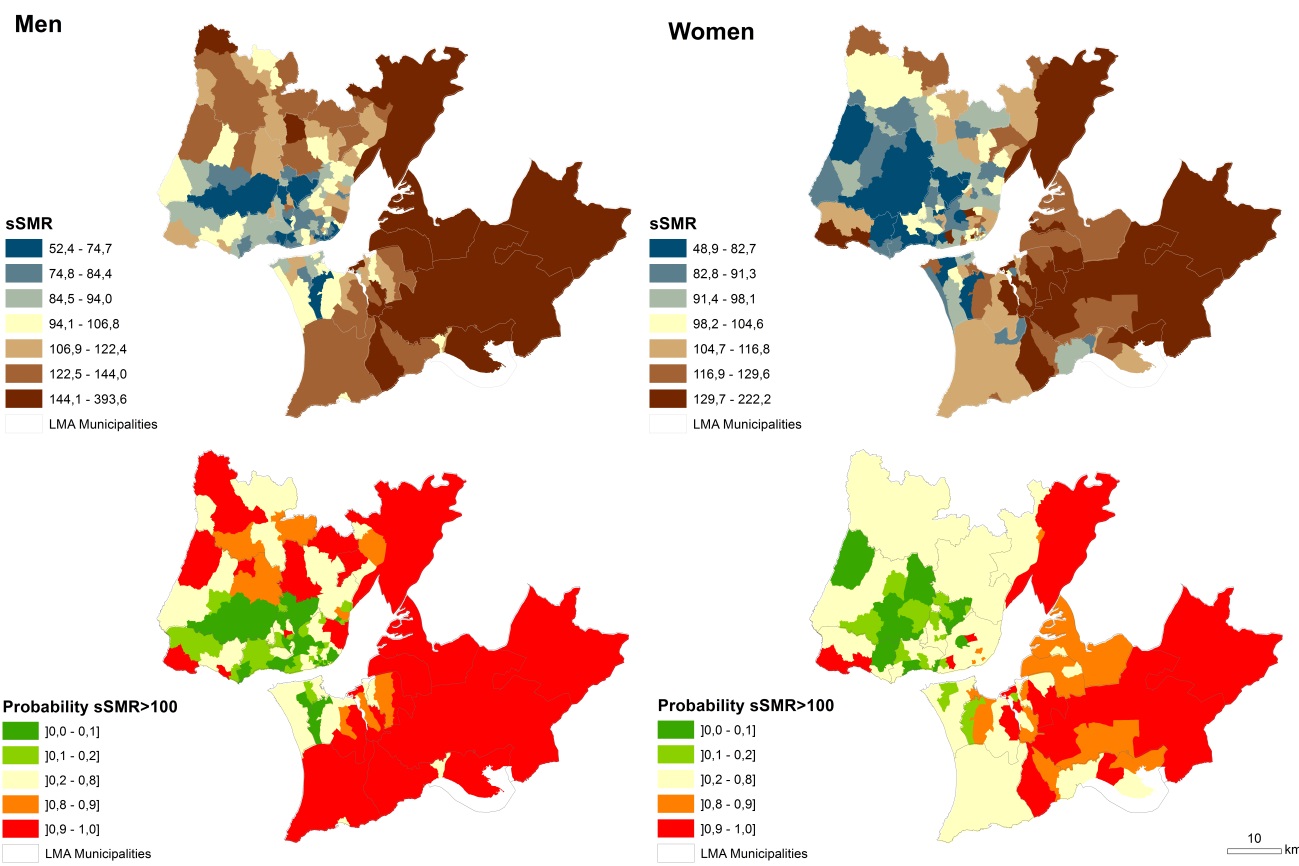


Source: based on Portuguese National Statistics Institute, 1995-2008.

Smoothed standardized mortality ratios (sSMR) for **Suicide and intentional self-harm** in LMA and the probability that the sSMR is higher than 100


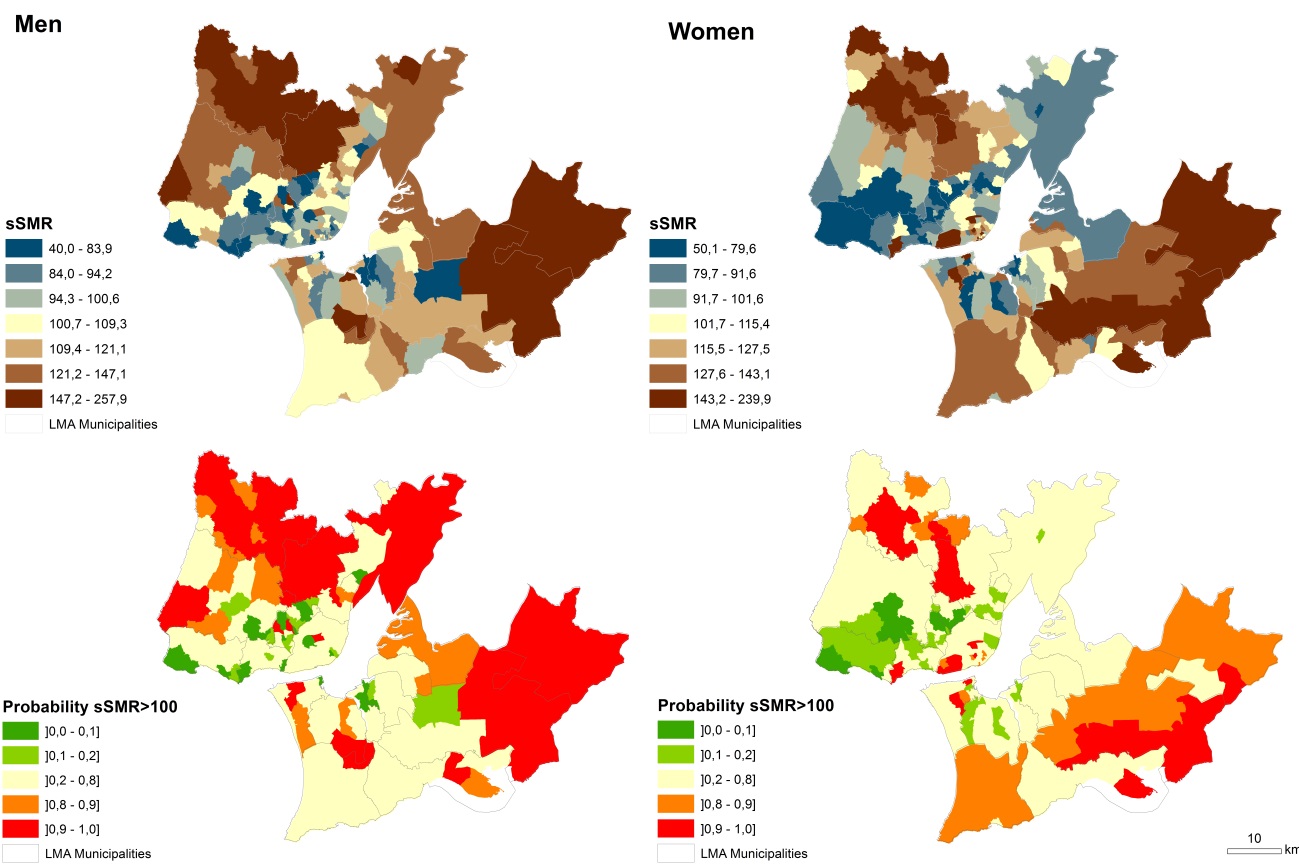


Source: based on Portuguese National Statistics Institute, 1995-2008.
